# Eosinophilic granulomatosis with polyangiitis with an abnormally sized nasal polyposis in a 24-year-old male: a case report

**DOI:** 10.1097/MS9.0000000000005301

**Published:** 2026-07-14

**Authors:** Kritika Bhattarai, Ashish Acharya, Santosh Basyal, Aashish Neupane, Shreema Basnet, Shobha Mandal, Jeevan Gautam, Anamol Acharya

**Affiliations:** a Department of Internal Medicine, Nepal Medical College and Teaching Hospital, Kathmandu, Nepal; b Department of Internal Medicine, Loyola Medicine MacNeal Hospital Program, Berwyn, Illinois, USA; c Department of Internal Medicine, New York Medical College/Lower Bucks Hospital Program, Bristol, Pennsylvania, USA; d Department of Internal Medicine, Tower Health Reading Hospital, West Reading, Pennsylvania, USA; e Department of Internal Medicine, St Agnes HealthCare Program, Baltimore, Maryland, USA; f Department of Internal Medicine, Parkview Health Program, Fort Wayne, Indiana, USA

**Keywords:** ANCA-associated vasculitis, case report, eosinophilic granulomatosis with polyangiitis, Nepal

## Abstract

**Introduction and importance:**

Eosinophilic granulomatosis with polyangiitis (EGPA) is a rare, multisystem vasculitic disorder characterized by asthma, eosinophilia, and necrotizing granulomatous inflammation with vasculitis affecting small- to medium-sized vessels. EGPA is classified as an antineutrophil cytoplasmic antibody (ANCA)-associated vasculitis.

**Presentation of case:**

Here, we present a case of a 24-year-old male who came to our institution with complaints of progressive, bilateral, non-tender, non-erythematous nasal swelling since last year. Upon further inquiry, he was found to have asthma managed with inhaled short-acting bronchodilators and had recurrent episodes of rhinosinusitis in the past. After several supportive investigations, a diagnosis of EGPA with nasal polyposis was confirmed, and the patient was initially treated with intravenous corticosteroids, followed by oral doses for maintenance therapy after discharge.

**Discussion:**

In most cases, EGPA presents clinically with a history of asthma. Upper respiratory tract involvement occurs in 70–90% of cases, typically manifesting as rhinosinusitis and nasal polyposis. A positive ANCA – especially with a perinuclear pattern (p-ANCA) and antigen specificity for myeloperoxidase (MPO-ANCA) – is a hallmark of EGPA and aids in diagnosis. Treatment generally includes high-dose corticosteroids, with cyclophosphamide added in severe cases.

**Conclusion:**

Diagnosing EGPA can be challenging due to its variable clinical manifestations. Our case exhibited typical upper respiratory tract involvement; however, positive ANCA, p-ANCA, and MPO-ANCA findings were instrumental in confirming the diagnosis. Based on our literature review, we recommend that such cases be thoroughly evaluated and managed in hospital settings.

## Introduction

Eosinophilic granulomatosis with polyangiitis (EGPA) is a rare multisystem disorder characterized by eosinophil-rich and necrotizing granulomatous inflammation, often involving the respiratory tract, and by necrotizing vasculitis that predominantly affects small- to medium-sized vessels. EGPA is typically associated with asthma and eosinophilia^[^1^]^. First identified in 1951 by pathologists Jacob Churg and Lotte Strauss, EGPA is also known as Churg–Strauss syndrome^[^2^]^. EGPA is strongly associated with anti-neutrophil cytoplasmic antibody (ANCA), with the disease entity falling under ANCA-associated vasculitis (AAV)^[^3^]^.

HIGHLIGHTS

- Eosinophilic granulomatosis with polyangiitis (EGPA) is an uncommon vasculitic disorder associated with asthma and eosinophilia, and falls under an antineutrophil cytoplasmic antibody (ANCA)-associated vasculitis.
- The majority of patients present with upper respiratory tract involvement, and the condition occurs in approximately 70–90% of cases, typically presenting with rhinosinusitis and nasal polyposis.
- A positive immunoassay for perinuclear-ANCA and myeloperoxidase ANCA is conducted to diagnose the case.
- High doses of corticosteroids, along with cyclophosphamide, are given as treatment.
- In our case report, we aim to present a case of EGPA in a young male with an unusual presentation of large polyposis and highlight the importance of managing such cases.

We have presented the case report in accordance with the CARE Guidelines^[^4^]^. The case report is compliant with the TITAN Guidelines 2025 – governing declaration and the use of AI^[^5^]^. Hence, no AI was used in this research paper.

## Case presentation

### History and physical examination

A 24-year-old unmarried Hindu male from Kathmandu, Nepal, presented with a chief complaint of progressive nasal swelling over the past year. He had a 5-year history of asthma, managed with inhaled short-acting bronchodilators. Before the presentation, the patient reported that his asthma was well-controlled with medications. The swelling was painless but gradually increased in size, eventually involving both alae of the nose and extending toward the upper lip and maxillary region of the face. The patient reported no previous history of similar swelling.

There was no history of epistaxis, anosmia, hyposmia, parosmia, shortness of breath, or cough at the time of presentation. Additionally, systemic symptoms such as rashes, joint swelling, joint pain, fatigue, headache, nausea, vomiting, and abdominal pain were absent. There was no history of ingestion or application of irritants or unwanted substances. His bowel and bladder habits were normal.

**Figure 1.**
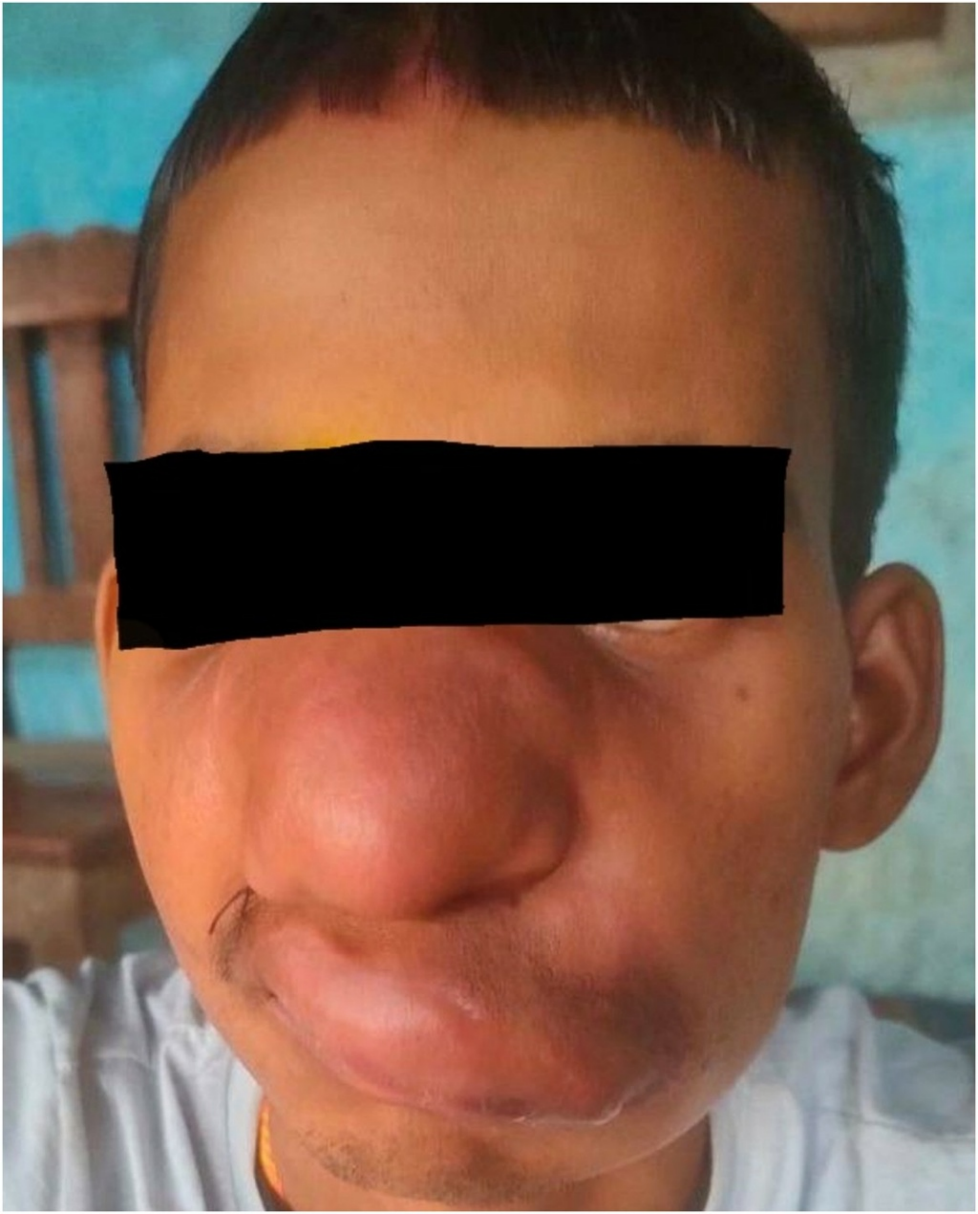
Gross image of the patient showing a diffuse, non-erythematous edema measuring approximately 4 × 5 cm, involving the bilateral nasal ala, with extension of the edema to the upper lip margin and the left maxillary region of the face.

On further inquiry, the patient reported a history of recurrent sinusitis, which had been managed medically. He had no significant family or surgical history. On examination, he was well-oriented to time, place, and person. His vitals and cardinal signs during the time of presentation were stable. On gross examination, a diffuse, non-erythematous, non-tender swelling measuring approximately 4 × 5 cm, involving the bilateral nasal ala with extension to the upper margin of the lip and left maxilla, was observed (Fig. 1).

The cardiorespiratory examination findings were within normal limits.

### Diagnosis and interpretations

The patient was subsequently admitted to the institution. Routine investigations, including a peripheral blood smear, revealed peripheral eosinophilia (24.2%) with normal hemoglobin and platelet counts. Liver function tests were within normal limits. Renal function tests showed serum urea and creatinine levels of 19 mg/dL and 0.8 mg/dL, respectively, with serum sodium at 141 mEq/L and potassium at 4.3 mEq/L, indicating normal renal function with no renal involvement. Urine analysis showed no casts, pus cells, or red blood cells.

However, inflammatory markers, such as C-reactive protein and erythrocyte sedimentation rate, were significantly elevated. Tests for tuberculosis and invasive fungal aspergillosis were negative.

Given the patient’s recurrent history of asthma and sinusitis, along with elevated inflammatory markers, an inflammatory disorder was suspected. Autoimmune serology revealed a raised antinuclear antibody titer of 1:160 and an elevated perinuclear-ANCA (p-ANCA) level of 65 U/mL, further supporting the diagnosis of AAV.

A contrast-enhanced computed tomography (CECT) of the paranasal sinuses demonstrated evidence of irregular soft tissue thickening involving the tip, bilateral ala of the nose, and bilateral anterior maxilla, with soft tissue opacification united in the right frontal and right ethmoidal air cells, indicating an abnormally sized nasal polyposis. A few enlarged lymph nodes were noted along the bilateral upper jugular chain, with the largest measuring 13 × 8 mm in size. Based on the physical examination and the CECT reports, the polyp was considered abnormally large.

A specimen from the right nasal vestibule was collected for histopathological examination and sent to another higher institution. The report revealed a biopsy specimen containing stratified squamous epithelium with eosinophilic infiltration of the stroma, along with characteristic fibrinoid necrosis of the vessel wall and multinucleated giant cells forming an ill-defined granuloma.

Based on the patient’s history, physical examination, and corroborating laboratory, radiological, and histopathological findings, a diagnosis of EGPA was established.

### Therapeutic interventions, discharge, and follow-up

Intravenous methylprednisolone was started as a line of treatment. He was discharged after a week’s stay in the hospital. As of now, the patient is maintained on oral steroids, and he has been visiting our institution for subsequent follow-ups regularly. The corticosteroid treatment did improve the polyp size by decreasing the inflammation and reducing the p-ANCA level on the follow-up visit. Despite the medical treatment, the polyp size warranted a surgical intervention for definitive management, for which the patient was referred to a higher center.

He informed us that he was fully satisfied with the received treatments and reported having no complications.

## Discussion

Churg–Strauss syndrome, or EGPA, a small vessel AAV, is a rare disease entity with an annual incidence ranging from 0.5 to 6.8 per million and a prevalence of 10.7–14 per million^[^1,6^]^. The exact incidence and patient distribution of EGPA in Nepal are unknown due to limited epidemiological data. EGPA was first diagnosed in 1990 using the classification system of the American College of Rheumatology^[^7^]^. It typically affects young adults in the age group of 40–60 years and develops in three sequential phases: (1) the allergic phase, characterized by asthma, allergic sinusitis, and rhinitis; (2) the eosinophilic phase, characterized by eosinophilic infiltrations in the organs, including the pulmonary, gastrointestinal, and cardiac systems; and (3) the vasculitic phase, distinguished by purpura, mononeuritis multiplex, and constitutional symptoms like fever, lethargy, fatigue, and weight loss^[^8^]^.

EGPA, along with granulomatosis with polyangiitis and microscopic polyangiitis, falls under the AAV^[^3^]^.

Asthma typically occurs in almost all cases, followed by the onset of vasculitis after several years. Upper airway involvement occurs in approximately 70–90% of cases, usually in the form of rhinosinusitis, often in the presence of nasal polyposis. Pulmonary involvement (70–90%) is seen as bilateral ground-glass pulmonary infiltrates. Extrapulmonary involvement, including constitutional features, musculoskeletal, cutaneous, cardiac, and peripheral nervous system manifestations, has also been reported^[^9^]^. Our case had characteristic upper respiratory tract involvement with a recurrent history of rhinosinusitis in the past and had now presented with abnormally enlarged nasal polyps. These symptoms of upper respiratory tract involvement, along with recently developed nasal polyps in the patient, were related to EGPA.

The diagnosis can be evidenced by performing an autoimmune screening test. Approximately 50% of patients are ANCA positive, most of whom exhibit a perinuclear pattern (p-ANCA) with antigen specificity for myeloperoxidase (MPO-ANCA)^[^10^]^.

We had some difficulties in diagnosing the case initially, as there were no cutaneous manifestations, and the lower lung field was clear on examination. Hence, we would like to highlight such an uncommon presentation of this disease entity in the existing literature.

A differential diagnosis for tuberculosis and fungal aspergillosis was made initially but was ruled out by the supporting investigations.

The Five Factor Score is a tool designed to assess the prognosis and diagnosis of systemic vasculitic conditions like EGPA. The 1996 FFS included the following parameters that were associated with poor prognosis: proteinuria >1 g/d, renal insufficiency, cardiomyopathy, severe gastrointestinal manifestations, and central nervous system involvement. For FFS values of 0, 1, and >2, the respective 5-year mortality rates were 12, 26, and 46%, respectively^[^11^]^.

A systematic review in 2016 reported that a variable 48–96% of patients with EGPA presented with head and neck manifestations, fulfilling the diagnosis as per the American College of Rheumatology^[^12^]^.

The initial management of EGPA includes high doses of corticosteroids, i.e., mg/kg/day of prednisone or its equivalent of methylprednisolone. The administration of methylprednisolone pulses (usually 15 mg/kg intravenously, repeated at 24-hour intervals for 1–3 days) can be used at the onset of therapy for the most severe forms. As soon as the patient improves and inflammatory markers return to normal (which usually takes 3 weeks to 1 month), corticosteroid-dose tapering can begin. Cyclophosphamide is used in combination with corticosteroids as a first-line treatment when there is a poor disease prognosis. An intravenous cyclophosphamide pulse of a dose of 600–750 mg/m^2^ is given at 2-week intervals for the first month and then at monthly intervals^[^6^]^. An additional therapy with mepolizumab (anti-IL-5 antibody), omalizumab (anti-IgE antibody), and rituximab (anti-CD20 antibody), which are all monoclonal antibodies, has also been reported to be effective^[^13^]^. Studies report that 95.8% of the cases were responsive to steroids and 60% of the patients required additional therapy^[^12^]^.

There is emerging literature on large language models in healthcare, which highlights their increasing influence on medical research, while simultaneously identifying important challenges related to validation and ethical use^[^14^]^. These considerations are relevant to this case report regarding the maintenance of strong standard clinical evidence.

## Conclusion

EGPA is a rare vasculitic disease condition that is often difficult to diagnose in a clinical setting but can be supported by specific laboratory, histopathological, and radiological investigations. Our case only had upper respiratory tract involvement, which made it difficult to ascertain the diagnosis but was later evidenced through the supportive work-up. The mainstay of management includes high doses of corticosteroids, as given in our case, with additional immunosuppressive drugs like cyclophosphamide in severe cases. Lastly, we would like to emphasize that such cases should be thoroughly worked up in a hospital setting, excluding other differential diagnoses.

## Data Availability

Not applicable.

## References

[1] Jennette JC Falk RJ Bacon PA. 2012 revised international chapel hill consensus conference nomenclature of vasculitides. Arthritis Rheum 2013;65:1–11. 23045170 10.1002/art.37715

[2] Churg J Strauss L. Allergic granulomatosis, allergic angiitis, and periarteritis nodosa. Am J Pathol 1951;27:277–301. 14819261 PMC1937314

[3] Mohammad AJ. An update on the epidemiology of ANCA-associated vasculitis. Rheumatology (Oxford) 2020;59:iii42–iii50. 32348522 10.1093/rheumatology/keaa089

[4] CARE checklist — CARE case report guidelines. CARE Case Report Guidelines. Accessed 9 July 2026. https://www.care-statement.org/checklist

[5] Agha R Mathew G Rashid R. Transparency in the reporting of Artificial INtelligence – the TITAN guideline. Prem J Sci 2025. doi:10.70389/pjs.100082

[6] Pagnoux C Guilpain P Guillevin L. Churg-Strauss syndrome. Curr Opin Rheumatol 2007;19:25–32. 17143092 10.1097/BOR.0b013e3280119854

[7] Masi AT Hunder GG Lie JT. The American college of rheumatology 1990 criteria for the classification of Churg-Strauss syndrome (allergic granulomatosis and angiitis). Arthritis Rheum 1990;33:1094–100. 2202307 10.1002/art.1780330806

[8] Mahmood K Butt NI Ashfaq F. Eosinophilic granulomatosis with polyangiitis (EGPA): a case report with atypical presentation. Pak J Med Sci 2023;39:307–09. 36694733 10.12669/pjms.39.1.6436 PMC9842979

[9] Taormina G Andolina G Banco MA. An uncommon presentation of eosinophilic granulomatosis with polyangiitis: a case report. J Med Case Rep 2014;8. doi:10.1186/1752-1947-8-190 PMC4086703 24928069

[10] Sinico RA Di Toma L Maggiore U. Renal involvement in Churg-Strauss syndrome. Am J Kidney Dis 2006;47:770–79. 16632015 10.1053/j.ajkd.2006.01.026

[11] Guillevin L Pagnoux C Seror R. The five-factor score revisited: assessment of prognoses of systemic necrotizing vasculitides based on the French Vasculitis Study Group (FVSG) cohort. Medicine (Baltimore) 2011;90:19–27. 21200183 10.1097/MD.0b013e318205a4c6

[12] Goldfarb JM Rabinowitz MR Basnyat S. Head and neck manifestations of eosinophilic granulomatosis with polyangiitis. Otolaryngol–Head Neck Surg 2016;155:771–78. 27352890 10.1177/0194599816657044

[13] Kimura Y Ito R Hayashi Y. A case of eosinophilic granulomatosis with polyangiitis showing multiple white lichen lesions on the airway mucosa. Respir Med Case Rep 2021;33:101451. 34401290 10.1016/j.rmcr.2021.101451 PMC8349094

[14] Guo SB Liu DY Fang XJ. Current concerns and future directions of large language model ChatGPT in medicine: a machine-learning-driven global-scale bibliometric analysis. Int J Surg 2026;112:2805–22. 41133425 10.1097/JS9.0000000000003668

